# Population genomics and conservation management of a declining tropical rodent

**DOI:** 10.1038/s41437-021-00418-9

**Published:** 2021-03-04

**Authors:** Brenton von Takach, Cara E. Penton, Brett P. Murphy, Ian J. Radford, Hugh F. Davies, Brydie M. Hill, Sam C. Banks

**Affiliations:** 1 Research Institute for the Environment and Livelihoods, Charles Darwin University, Darwin, Northern Territory Australia; 2 NESP Threatened Species Recovery Hub, Research Institute for the Environment and Livelihoods, Charles Darwin University, Darwin, Northern Territory Australia; 3 Department of Biodiversity, Conservation and Attractions, Kununurra, WA Australia; 4 Flora and Fauna Division, Department of Environment, Parks and Water Security, Northern Territory Government, Darwin, Northern Territory Australia

**Keywords:** Ecological genetics, Population genetics, Conservation biology, Biogeography

## Abstract

Conservation management is improved by incorporating information about the spatial distribution of population genetic diversity into planning strategies. Northern Australia is the location of some of the world’s most severe ongoing declines of endemic mammal species, yet we have little genetic information from this regional mammal assemblage to inform a genetic perspective on conservation assessment and planning. We used next-generation sequencing data from remnant populations of the threatened brush-tailed rabbit-rat (*Conilurus penicillatus*) to compare patterns of genomic diversity and differentiation across the landscape and investigate standardised hierarchical genomic diversity metrics to better understand brush-tailed rabbit-rat population genomic structure. We found strong population structuring, with high levels of differentiation between populations (*F*_ST_ = 0.21–0.78). Two distinct genomic lineages between the Tiwi Islands and mainland are also present. Prioritisation analysis showed that one population in both lineages would need to be conserved to retain at least ~80% of alleles for the species. Analysis of standardised genomic diversity metrics showed that approximately half of the total diversity occurs among lineages (*δ* = 0.091 from grand total *γ* = 0.184). We suggest that a focus on conserving remnant island populations may not be appropriate for the preservation of species-level genomic diversity and adaptive potential, as these populations represent a small component of the total diversity and a narrow subset of the environmental conditions in which the species occurs. We also highlight the importance of considering both genomic and ecological differentiation between source and receiving populations when considering translocations for conservation purposes.

## Introduction

To conserve threatened species, conservation biologists have historically been required to make decisions with basic, and often inadequate, information (Soulé 1985). We now know that management actions such as ecological restoration and translocation programs benefit when information on population genetic structure and genetic diversity is incorporated into strategic planning (Frankham 2005; Frankham et al. 2014). The recent explosion in low-cost next-generation sequencing platforms has made such information routinely available to conservation managers, enabling the inclusion of genetic data in the development of conservation management strategies (Ralls et al. 2018).

The spatial distribution of genetic diversity is largely driven by patterns of gene flow, genetic drift and local adaptation (Orsini et al. 2013). Unfortunately, historical patterns of population genetic structure are being disrupted by anthropogenic influences on ecosystems. The increasing isolation and fragmentation of populations and habitats is contributing to a reduction in genetic diversity, increasing the potential for inbreeding depression as well as the risk of extinction (Saccheri et al. 1998; Storck-Tonon and Peres 2017; Frankham et al. 2019). For many declining species, the loss of genetic diversity may be a secondary threatening process whose effects are brought on by the demographic impacts of primary threatening processes (Caughley 1994). Therefore, in ecosystems where species are in severe decline, genetic diversity is likely to be an important consideration when planning for the conservation and recovery of threatened species (Ottewell et al. 2016). Genetic analyses provide an improved understanding of the taxonomic identity of populations and species, assessments of the loss of genetic diversity from populations of threatened species and guidelines for genetic rescue, reintroduction or translocation strategies.

Australia has one of the worst mammal extinction records of any country over the past 200 years (Woinarski et al. 2019), and the northern part of the continent, widely considered ecologically ‘intact', is currently experiencing a drastic decline of its native mammal fauna, most likely due to habitat degradation (Legge et al. 2011; Stobo-Wilson et al. 2020), introduced species (Stobo‐Wilson et al. 2020; Radford et al. 2020), inappropriate fire regimes (Woinarski et al. 2011; von Takach et al. 2020) and a changing climate (Kutt et al. 2009; Traill et al. 2011). In northern Australia, there have been very few range-wide studies of population genetics using next-generation sequencing with large genome-wide single-nucleotide polymorphism (SNP) datasets. The northern Australian mammal declines are likely to be causing the loss of considerable amounts of genetic diversity and adaptive potential, with resulting losses in the ability of species to recolonise large areas of their former ecological niche (Scheele et al. 2017; Doherty and Driscoll 2018).

Australia contains a diverse array of native rodents in the subfamily Murinae (Aplin 2006; Breed and Ford 2007), with 67 species of rodents in 34 genera thought to be extant prior to colonisation by Europeans (Roycroft et al. 2020). The genus *Conilurus* is one of the so-called ‘old endemic' rodents that colonised continental Australia from New Guinea between 6 and 9 million years ago, with three species of *Conilurus* historically present in Australia. These include the extant brush-tailed rabbit-rat (*C. penicillatus*), and the extinct white-footed rabbit-rat (*C. albipes*) and the Capricorn rabbit-rat (*C. capricornensis*) (Cramb and Hocknull 2010; Woinarski et al. 2019). The brush-tailed rabbit-rat is known from both Australia and Papua New Guinea (PNG), although very little is known about the species in PNG. Tentative subspecies have been established based on morphological analysis, consisting of two Australian subspecies (*C. p. melibius*, on the Tiwi Islands and *C. p. penicillatus*, all other Australian populations) and a single PNG subspecies (*C. p. randi*) (Kemper and Schmitt 1992). The brush-tailed rabbit-rat has undergone a rapid decline in the last half-century, with a recent study showing that it has experienced the greatest reduction in the extent of occurrence of nine declining savanna mammal species (von Takach et al. 2020). As a result of these declines, the species has been listed as Vulnerable under both Australian national legislation and by the IUCN. The most intact populations of the brush-tailed rabbit-rat now persist on large islands and peninsulas along the northern Australian coast, although there are signs of decline in these areas as well (Davies et al. 2017; Davies et al. 2018). The reasons for its decline remain uncertain, although predation by feral cats, possibly exacerbated by inappropriate fire regimes (frequent, high-intensity fires that reduce denning and ground cover resources) and grazing by feral herbivores (e.g. buffalo and cattle) are strongly implicated (Firth et al. 2010; Davies et al. 2017, 2020; Stobo-Wilson et al. 2020).

With many of the Earth’s species disappearing from parts of their ranges, translocations are being increasingly used to restore locally extinct populations (Silcock et al. 2019). Many of these translocations are inadequately designed to maximise the likelihood of success and do not address the ultimate drivers of decline or the presence of multiple conservation management units (Palsbøll et al. 2007; Pérez et al. 2012). As a lack of baseline knowledge is reported in 22% of reintroductions (Berger-Tal et al. 2020), it is critical that we increase the amount and availability of information regarding the population genetic structure in declining species. This will help to inform the most appropriate source populations for translocation, and can even identify the proportion of the available genetic diversity and thus adaptive potential, that is represented in the translocated population.

Here, we use the brush-tailed rabbit-rat as a case study to explore current patterns of genetic diversity across the isolated remnant populations of this formerly widespread and abundant native mammal (Woinarski et al. 2001), and consider the consequences of this diversity for the long-term conservation and recovery of the species. We quantify genetic variation within and among major extant populations of the species using typical genomic diversity parameters as well as a standardised diversity metric Q (derived from Rao’s Quadratic Entropy) that partitions genomic diversity into hierarchical strata (Smouse et al. 2017). Formal conservation prioritisation analysis allows us to identify optimal approaches for conserving genomic diversity in the species (Petit et al. 1998; Hanson, Schuster et al. 2020). These findings are then built upon through ecological niche hypervolume and overlap analysis (Blonder et al. 2018), to quantify differences in environmental conditions across the range of major lineages.

We hypothesise that the brush-tailed rabbit-rat will show a high level of genetic differentiation between regions, suggesting that multiple populations or management units require separate conservation management actions in order to conserve high levels of allelic diversity and adaptive capacity. We further predict that strong differences in environmental conditions of the realised niche between major lineages will be present, due to the species presence on climatically unique offshore islands as well as the large (~800 km) geographic distances between some mainland populations. We expect that such findings will (1) help confirm the presence of two Australian subspecies of brush-tailed rabbit-rats, (2) have consequences for the conservation management of the brush-tailed rabbit-rat as well as other species in the region and (3) showcase how the success or failure of conservation efforts to protect this flagship species is likely to inform future management of small- to medium-sized mammals in northern Australia.

## Materials and methods

### Sample collection

**Fig. 1:**
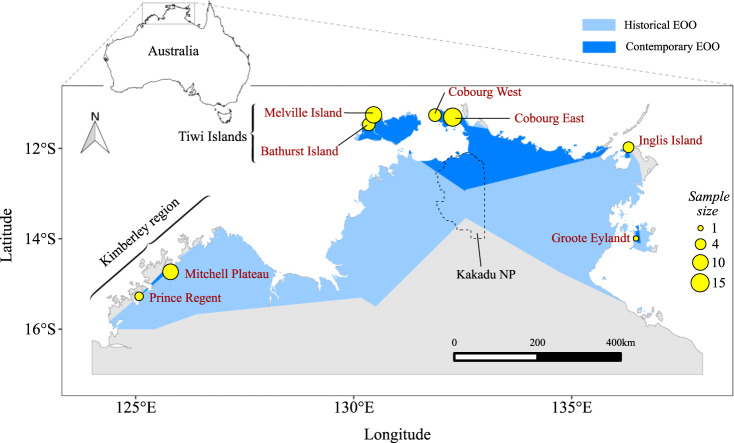
Map of north-western Australia showing the locations (yellow circles) of brush-tailed rabbit-rat (*Conilurus penicillatus*) tissue samples collected for population genomics analyses, with points sized relative to the number of samples collected from each population. Blue shading represents the historical (light blue, prior to 2000) and contemporary (dark blue, 2000 onwards) extents of occurrence (EOO) of the species, as defined by *α*-hull polygons of all available records in the Atlas of Living Australia (http://www.ala.org.au/). The boundary of Kakadu National Park is shown as a dotted line, with the species now thought to be extinct from that region.

We obtained 55 tissue samples from multiple research and/or monitoring groups who had trapped mammals, primarily between 2005 and 2019. These samples were typically collected during fieldwork, with a small section of ear tissue taken from live animals and placed into a vial containing either ethanol or dimethyl sulfoxide solution. In addition, six liver samples were obtained from the Australian Biological Tissue Collection of the South Australian Museum, which stored specimens that were collected in 1981/82 from the Mitchell Plateau in Western Australia. Together, this produced a total of 61 tissue samples. These samples covered a large proportion of the species’ extant range (Fig. 1). The species is believed to have been extirpated from Kakadu National Park, a major World Heritage-listed park in the Northern Territory, sometime in the 2000s (Woinarski et al. 2014) and no samples were available from this location. While samples were collected over slightly different geographic scales between regions (due to differing animal densities and trapping layouts), they were collected at eight localities, of which four were located on islands and four on the Australian mainland. Sample sizes ranged from 1 to 15 per locality (Fig. 1), with five localities having ≥7 samples (Mitchell Plateau, Cobourg East, Cobourg West, Melville Island and Bathurst Island) available and three localities (Groote Eylandt, Prince Regent and Inglis Island) having <7 samples available.

### DNA extraction and sequencing

Tissue samples were prepared and extracted in plate format (Qiagen DNeasy 96 Blood & Tissue Kit) following the standard protocol, with lysis consisting of 2 h incubation at 56 °C followed by overnight incubation at 37 °C. Extractions were then quantified using a broad-range dsDNA assay kit for the Qubit Fluorometer 3.0 and normalised to 200 ng in 25 µL for library preparation. A single 96-well plate, consisting of our 61 samples and 34 technical replicates, was sent to the Australian Genome Research Facility (AGRF) in Melbourne, Victoria, for double-digest restriction-associated DNA (ddRAD) sequencing (Peterson et al. 2012). Three samples were used by AGRF to establish the optimal combination of restriction enzymes, with PstI and HpyCH4IV considered most suitable for minimising repetitive sequences and maximising amplification. These three samples were broadly representative of the species distribution, including the Mitchell Plateau, Bathurst Island and Cobourg Peninsula. Briefly, the library preparation protocol consisted of (1) digestion using PstI and HpyCH4IV, (2) ligation with one of 48 unique inline barcoded adapters compatible with the restriction site overhang, (3) manual sample pooling, (4) DNA purification (QIAquick PCR Purification Kit followed by SPRIselect paramagnetic beads), (5) size-selection targeting fragments of 280–375 bp in size (BluePippin, Sage Science) and (6) a PCR amplification step where one of two multiplexing index primers was added. Indexed libraries were then pooled together and loaded onto a high-output flowcell for 150-bp single-end sequencing across four lanes of an Illumina NextSeq 500 platform.

### Bioinformatic pipeline and SNP filtering

We obtained 403.6 million raw sequence reads from the ddRAD sequencing, which was demultiplexed and trimmed to 125 bp (phred quality score ≥30) via the *process_radtags* function of the stacks software package (Catchen et al. 2013), retaining 398.2 million reads. Reads were aligned to the *Mastacomys fuscus* chromosome-length genome assembly (https://www.dnazoo.org/assemblies/Mastacomys_fuscus) using the bwa (v0.7.17) *mem* algorithm (Li 2013), with the resulting sequence alignment/map (SAM) file for each individual converted to binary alignment/map (BAM) files using samtools v1.7-1 (Li et al. 2009). Also using samtools, unmapped reads were then filtered out of each BAM file, and reads were sorted by scaffold number and position, and the BAM file indexed. The angsd v0.93 software package (Korneliussen et al. 2014) was then used to perform initial filtering to identify single-nucleotide polymorphisms (SNPs) and create a SNP-by-sample matrix. Parameters used in angsd were similar to those used in von Takach Dukai et al. 2020, with reads only used to call SNPs if the map quality was ≥20 (thus excluding repeat regions), and loci only retained if they were polymorphic (likelihood ratio test *P* value ≤1 × 10^−5^), were genotyped in at least 50% of individuals, had a minimum of five reads per locus per sample and had fewer than 1000 reads per locus per sample. Genotypes were called using posterior probabilities assuming a uniform prior, with a posterior probability threshold of at least 0.98 (using GATK genotype likelihoods). This retained a total of 225,646 SNPs, with a mean read depth per SNP per sample of 33.9, and a median read depth per SNP per sample of 22.7. The SNP by sample matrix was then read into the statistical analysis software R v4.0.0 (R Core Team 2020) for all remaining analyses. Filters applied in base R included removal of SNPs (1) where the proportion of samples in which loci were genotyped was less than 80%, (2) that had a minor allele count of less than three and (3) that had observed heterozygosity (*H*_O_) > 0.5. To remove SNPs in linkage disequilibrium, we used the *snpgdsLDpruning* function of the ‘SNPRelate’ package (Zheng et al. 2012). One of each SNP pair was removed if they had a correlation of >0.5 within a sliding window of 100,000 base pairs, which is the distance at which the 95th percentile of *r*^2^ in wild mice (*Mus musculus*) falls to less than 0.4 (Laurie et al. 2007). Finally, samples missing more than 25% of genotype calls were removed, resulting in the retention of 11,478 SNPs and 51 unique samples for analysis. The single sample from Groote Eylandt failed to produce useful sequence data due to DNA quality and/or quantity, so this locality was not investigated any further. To ensure that relationships between individuals and populations could be accurately inferred from the dataset, we produced a hierarchical clustering dendrogram based on genetic distance, with a visual examination of the dendrogram confirming that technical replicates paired closely together (Supplementary Fig. S1). The overall level of missing data for the filtered SNP by sample matrix was 1.9%.

### Geographic patterns of population differentiation

We visualised population genetic structure using a principal coordinate plot. Pairwise population Nei’s genetic distances were calculated using the *df2genind* and *dist.genpop* functions in the ‘adegenet’ (Jombart 2008) package. The first two principal coordinate dimensions of the resulting distance matrix were created using the *cmdscale* function, and populations plotted on the coordinates. The percentage of variance explained by each axis was also recorded. An individual-level principal coordinates analysis was also conducted, with genetic distances calculated using the *prevosti.dist* function of the ‘poppr’ package (Kamvar et al. 2014). To quantify the level of allelic isolation between populations, we calculated pairwise Nei genetic differentiation (*F*_ST_) using the *genet.dist* function of the ‘hierfstat’ package (Goudet 2005), as well as a standardised differentiation measure (*G*″_ST_) via the *pairwise_Gst_Hedrick* function of the ‘mmod’ package (Winter 2012). To determine whether sampling of closely related individuals was likely to be influencing our results, we also calculated the *F*_ST_ and *G*″_ST_ values after removing one of any pair of closely related individuals in the dataset. To estimate pairwise kinship coefficients between individuals within sampling localities, we used two methods: (1) the *snpgdsIBDMLE* function of the ‘SNPRelate’ package, and (2) the *beta.dosage* function of the ‘hierfstat’ package. Pairwise coefficients with values of either >0.125 (SNPRelate) or >0.25 (hierfstat) were considered closely related.

To identify patterns of hierarchical population structuring, we used the cross-entropy methods of the ‘LEA’ package (Frichot, François 2015). This package applies a model of genetic structure featuring a discrete number (*K*) of ancestral populations, allowing for the independent investigation of values for *K* that have low cross-entropy metrics (Frichot et al. 2014). Cross-entropy criteria were calculated for values of *K* between one and 10, and a cross-entropy scree-plot was output for visual interpretation. The matrices of individual admixture coefficients were then extracted and plotted as stacked bar plots to visualise the hierarchical population structure. As above, this was done both before and after removing closely related individuals, to identify whether inbred individuals or sampling of family groups were likely to influence our analysis of population genomic structure.

Isolation-by-distance of all mainland individuals (i.e. excluding individuals from islands) was investigated using spatial autocorrelation of multilocus genotypes, plotted on a correlogram. Pairwise geographic distances between individuals were calculated from latitude and longitude observations for each sample via the *earth.dist* function of the ‘fossil’ package (Vavrek 2011). Correlation and significance values were made using the *genetic_autocorrelation* function of the ‘gstudio’ package (Dyer 2016) with 999 permutations, and the results plotted in R.

### Population genomic diversity

We calculated mean values of standard genomic diversity parameters for each population with at least six individuals, including the number of alleles (*A*), the number of effective alleles (*A*_E_), observed heterozygosity (*H*_O_), expected heterozygosity (*H*_E_), Wright’s inbreeding coefficient (*F*_IS_) and the locus polymorphic index (*P*_E_). All calculations were made using the ‘gstudio’ package (Dyer 2016) with a small sample-size correction. These diversity parameters were also calculated after the removal of any closely related individuals, to determine whether this was likely to be influencing the patterns of heterozygosity or inbreeding coefficients.

To investigate the partitioning of genetic diversity among and within geographic regions and individual populations, we used the *QDiver* function (Smouse et al. 2017) in the GenAlEx v6.51b2 (Peakall and Smouse 2006, 2012) software package. This method computes standardised genetic diversity metrics partitioned into hierarchical strata, and allows for evaluation of homo-/heterogeneity of within-stratum diversity components (Smouse et al. 2017). The Q metric used by *QDiver* is derived from Rao’s Quadratic Entropy, a commonly used measure of diversity in ecological communities, with values converted into a diversity analogue suitable for genomic data. The function creates a ‘diversity cascade’ that includes the total diversity of the species (γ), the diversity among lineages/regions (*δ*), the within-region/lineage diversity (σ), the among-population diversity within each region/lineage (β) and the within-population diversity within each region/lineage (α). As this function does not yet allow for missing data, we removed any SNPs that were missing genotypes for any individual, retaining 6569 SNPs.

### Prioritising populations for the conservation of genomic diversity

We conducted a set of analyses to inform strategies for the conservation of genetic diversity in the species. First, we used the approach of Petit et al. (1998) to quantify the contribution of each population to the total allelic richness represented across the 11,478 SNP panel and the entire set of populations sampled. We combined the two Kimberley region populations (Mitchell Plateau and Prince Regent River) due to the low sample size in the latter population and used the *allel.rich* function of the ‘PopGenReport’ package (Adamack and Gruber 2014) to estimate mean allelic richness per locus over the entire dataset, standardised to a sample size of eight alleles to account for differences in sample sizes. We then iteratively removed each population from the dataset to estimate the proportional loss of allelic richness that would result from the extinction of any one of these populations. This was done using the formula AR(*t*)—AR(− *i*)/(AR(*t*) − 1), where AR(*t*) is total allelic richness and AR(−*i*) is allelic richness over all populations excluding the one in question.

Second, we used a marxan (Ball et al. 2009; Watts et al. 2009) analysis to identify networks of extant populations that best represent the total genetic diversity in the species, as estimated by the sampled populations across all SNP loci. In the absence of specific costed conservation options, we allocated an equal unit cost of 1 to conserve each population and identified the optimal network of populations to maximise allelic richness in the species, identifying optimal solutions for scenarios of 1, 2, 3, 4 or 5 ‘protected’ populations using the R package ‘prioritizr’ (Hanson, Schuster et al. 2020) and the symphony integer linear programming solver (Vladislav 2018). For each of 100 iterations, we randomly sampled 4 individuals per population, calculated allelic richness and the total number of alleles across all populations combined and identified a conservation solution for maximum coverage (of alleles) objective for budgets of 1, 2, 3, 4 and 5. We tallied the number of configurations across the 100 replicates for each budget, as well as the resulting allelic richness and total allele count for each solution.

### Niche hypervolume analysis

To assess the level of similarity in the environmental niche occupied by major lineages in the species, we investigated *n*-dimensional niche hypervolumes (Blonder et al. 2018). For each of the ancestral populations (identified by the most suitable number in the cross-entropy plot, described above), we calculated the niche hypervolume of five environmental variables that have previously been shown to be important correlates of brush-tailed rabbit-rat distribution (von Takach et al. 2020). These included three climatic variables (mean annual rainfall, mean maximum temperature of the hottest month and mean minimum temperature of the coldest month) taken from the SILO database (https://www.longpaddock.qld.gov.au/silo/), and two topographic variables, elevation (three arcsecond digital elevation model, obtained from Geoscience Australia http://www.ga.gov.au/) and ruggedness (derived from the digital elevation model in R, as detailed in von Takach et al. 2020). Values at each sampling location of a genotyped individual were extracted from raster layers, and niche hypervolumes for each ancestral population were calculated using the *hypervolume_svm* function of the ‘hypervolume’ package (Blonder and Harris 2018) in R. We then calculated the percentage overlap (Jaccard and Sorenson similarity metrics) between hypervolumes using the *hypervolume_overlap_statistics* function.

## Results

### Geographic patterns of population differentiation

We found high levels of genomic differentiation between populations, demonstrated by very high pairwise population *F*_ST_ (Table 1 and Supplementary Table S1) and *G*″_ST_ (Supplementary Tables S2 and S3) values, suggesting strong isolation of alleles due to low levels of recent gene flow. There was considerable genomic isolation (*F*_ST_ ≥ 0.6) between the Tiwi Islands (Melville and Bathurst populations) and all other populations, including the nearby Cobourg Peninsula (Table 1). The most geographically distant pairwise combination of the four mainland populations, Cobourg East and the Mitchell Plateau (separated by ~800 km), was only moderately differentiated in comparison (*F*_ST_ = 0.29). The first axis of the principal coordinate plot accounted for 87.1% of the total variation and separated the Tiwi Islands from the remaining populations, with the mainland and Inglis Island populations spreading across the second axis (Supplementary Figs. S2 and S3).

**Table 1:** Pairwise population genomic differentiation (*F*_ST_) between all four populations of the brush-tailed rabbit-rat (*Conilurus penicillatus*) with *n* ≥ 6.

	Cobourg East	Bathurst Island	Melville Island	Mitchell Plateau
Cobourg East (*n* = 15)	0			
Bathurst Island (*n* = 6)	0.72	0		
Melville Island (*n* = 11)	0.60	0.21	0	
Mitchell Plateau (*n* = 10)	0.29	0.78	0.66	0

All positive *F*_ST_ values are significant at *P* ≤ 0.001.

**Fig. 2:**
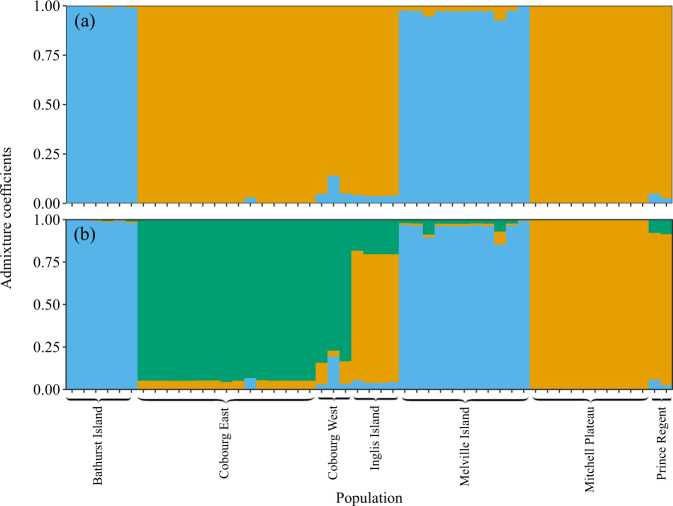
Patterns of population structuring in the brush-tailed rabbit-rat (*Conilurus penicillatus*). Panels (**a**) and (**b**) show the individual admixture coefficients when two or three ancestral genomic clusters are identified, respectively.

The cross-entropy plot from the LEA analysis of genetic structure showed decreasing cross-validation scores with increasing values of *K*, consistent with a hierarchical population structure, although there was a large drop between *K* = 1 and *K* = 2 (Supplementary Fig. S4), suggesting that there were two main ancestral components in the dataset (Fig. 2a). Visualisation of the admixture coefficients for *K* = 3 showed the Kimberley populations (Mitchell Plateau and Prince Regent) in the far west separating from the two Cobourg Peninsula populations, with the individuals on Inglis Island (in the far east of the species distribution) showing a mix of Cobourg and Kimberley ancestry (Fig. 2b). The observed population structuring did not change substantially with the removal of closely related individuals (Supplementary Fig. S5).

Analysis of spatial autocorrelation of genotypes identified significant values of the autocorrelation coefficient *r* persisting for distances exceeding 100 km (Supplementary Fig. S6), although there was a substantial reduction in *r* between 1 and 5 km.

### Population genomic diversity

Investigation of standard genomic diversity parameters showed some variation between populations (Supplementary Table S4). The effective number of alleles was the highest in the Melville Island population (*A*_E_ = 1.17) and lowest in the Bathurst Island population (*A*_E_ = 1.09). A similar trend was also present for expected heterozygosity, observed heterozygosity, Wright’s inbreeding coefficient and the locus polymorphic index. The mean observed heterozygosity across populations was 0.08, with a value of 0.06 for the Bathurst Island and Mitchell Plateau populations, and a value of 0.09 for the Cobourg East and Melville Island populations. One population, Melville Island, showed a much higher level of homozygote excess (*F*_IS_ = 0.31) compared to the other populations (*F*_IS_ = 0.02–0.03). While heterozygosity increased for the Melville Island population when closely related individuals were removed, the high *F*_IS_ did not dissipate (Supplementary Table S5).

The QDiver analysis allowed us to partition genetic diversity into hierarchical strata via a standardised diversity metric (Table 2), allowing for comparisons among and within strata (Smouse et al. 2017). We found that approximately half of the genetic diversity is found between the Tiwi Islands lineage and the mainland lineage (among regions *δ* = 0.091 from grand total *γ* = 0.184). The Tiwi Islands lineage (*σ* = 0.117) contained more diversity than the mainland lineage (*σ* = 0.099), although Bartlett’s test for homogenous within-region diversity suggested that the difference was not significant (*P* = 0.378). However, the mainland lineage (*β* = 0.014) did have a significantly (*P* = 0.001) greater level of among-population diversity (Mitchell Plateau vs Cobourg East) than the Tiwi Islands lineage (*β* = 0.008, Bathurst Island vs Melville Island). Within populations, Cobourg East (*α* = 0.094) had a greater level of diversity than the Mitchell Plateau (*α* = 0.06), and Melville Island (*α* = 0.121) had a greater level of diversity than Bathurst Island (*α* = 0.056).

**Table 2:** Diversity cascade for brush-tailed rabbit-rats, showing the amount of genetic diversity held within and among hierarchical strata.

Parameter	Lineage	Population	Diversity value
*γ* = *Q*(GT)			0.184
*δ* = *Q*(AR)			0.091
*σ* = *Q*(WR)	Mainland		0.097
	Tiwi Islands		0.113
*β* = *Q*(AP/WR)	Mainland		0.014
	Tiwi Islands		0.008
*α* = *Q*(WP/WR)	Mainland	Cobourg East	0.094
		Mitchell Plateau	0.060
	Tiwi Islands	Bathurst Island	0.056
		Melville Island	0.121

*GT* grand total, *AR* among region/lineage, *WR* within region, *AP/WR* among population within region, *WP/WR* within population within region.

Two populations on the Australian mainland and two populations on the Tiwi Islands were used in the analysis. The *Q* diversity value is derived from Rao’s Quadratic Entropy, a commonly used measure of diversity in ecological communities, with values converted into an analogue suitable for genomic data.

### Prioritising populations for the conservation of genomic diversity

Quantifying the contribution that each a priori population grouping made to the overall allelic richness of the species showed that Melville Island had the greatest unique contribution of alleles (0.12) (Table 3), followed by Cobourg and Bathurst Island (both 0.04). Inglis Island (0.01) and the Kimberley populations (−0.01) both had very small unique contributions of alleles, with the inclusion of the Kimberley populations marginally reducing overall AR. This agrees with the QDiver results, with the Cobourg, Kimberley and Inglis Island populations all part of the same greater lineage and thus sharing a considerable amount of allelic diversity. Although the diversity within each of these populations is high, there is also some redundancy in genetic diversity among them.

**Table 3:** Allelic richness (AR) of each a priori population grouping of the brush-tailed rabbit-rat, and the unique contribution that each group makes to the total allelic richness of the species (i.e. all genotyped individuals considered as one ‘population’).

	Cobourg	Bathurst Island	Melville Island	Inglis Island	Kimberley
Mean AR	1.31	1.14	1.34	1.08	1.17
SD	0.32	0.32	0.34	0.24	0.32
AR contribution	0.04	0.04	0.12	0.01	−0.01

**Fig. 3:**
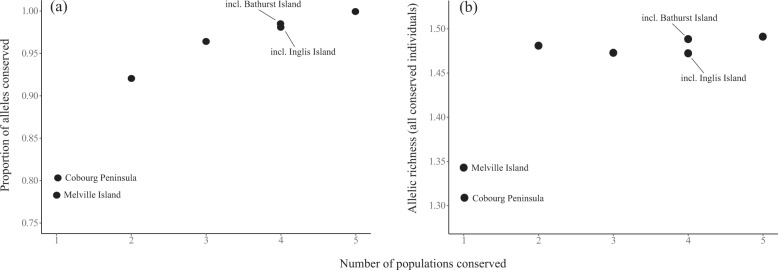
Optimisation scenarios for the brush-tailed rabbit-rat. Impacts of conserving differing numbers of a priori population groupings for the brush-tailed rabbit-rat on (**a**) the proportion of total alleles conserved and (**b**) allelic richness. The number of populations conserved in each scenario corresponds with those in Table 4. Where more than one population was identified as an optimal choice for conservation by the iterative marxan analysis, both scenarios are presented and the relevant populations are shown and labelled.

Given an equal cost for conserving each a priori population grouping, marxan suggested that Cobourg (52% of iterations) was the single most cost-effective region to conserve for allelic diversity in the brush-tailed rabbit-rat (representing ~80% of the alleles detected in the species), although the conservation benefit from Melville Island (48% of iterations) was very similar (~78% of alleles detected in the species) (Fig. 3). If two populations were to be conserved, Cobourg and Melville Island were always selected (Table 4). If three populations were conserved, the Kimberley was always selected. If four populations were conserved, Bathurst Island was usually (94% of iterations) selected, although Inglis Island was occasionally chosen (6% of iterations) instead. Conserving the populations of both Cobourg and Melville Island ensured that >90% of alleles across the species were conserved, with diminishing returns as additional populations are added to the hypothetical reserve system (Fig. 3). Conserving just one of these populations resulted in the loss of 10–15% of alleles. Similarly, allelic richness is substantially increased by conserving both Cobourg and Melville (AR = 1.48), with much lower allelic richness if a single population is conserved (Cobourg = 1.31; Melville = 1.34).

**Table 4:** Optimisation scenarios for conservation of alleles in a priori population groupings of the brush-tailed rabbit–rat.

Number of populations conserved	Cobourg	Bathurst Island	Melville Island	Inglis Island	Kimberley
1	0.52	0	0.48	0	0
2	1	0	1	0	0
3	1	0	1	0	1
4	1	0.94	1	0.06	1
5	1	1	1	1	1

Numbers show the proportion of times that each population was chosen in a given scenario from 100 iterations of randomly sampling four individuals per population and counting the number of alleles.

### Niche hypervolume analysis

We found that the Tiwi Islands lineage of the brush-tailed rabbit-rat had a substantially smaller niche hypervolume (2.88 × 10^−5^) than the mainland lineage (3.53 × 10^−2^), suggesting that the Tiwi Island individuals persist in a much more restricted set of environmental conditions. We also found no overlap in niche hypervolumes between the two lineages, based on either the Jaccard or Sorenson similarity metrics. This indicates that the environmental conditions occupied by the two lineages are differentiated, with neither niche being a complete or partial subset of the other. Pairwise plots of each niche dimension showed that the niche centroid of mean annual rainfall is substantially different between the two lineages (Supplementary Fig. S7).

## Discussion

Conservation management benefits greatly from incorporating information about the spatial distribution of genomic diversity into the planning process. Importantly, environmental or distance-based substitutes for genomic diversity metrics are not necessarily adequate (Hanson, Veríssimo et al. 2020). Quantification of geographic patterns of population genomic structure is therefore critically important to develop effective conservation management strategies for threatened species. Here, we found that a species of high conservation concern, the brush-tailed rabbit-rat, is characterised by high levels of genomic differentiation between populations in Australia, with a distinct lineage represented on two offshore islands (Bathurst and Melville). Investigation of ecological niche hypervolumes, combined with conservation prioritisation analysis of allelic diversity, showed that the two dominant lineages of the brush-tailed rabbit-rat occupy very different environmental conditions and that both would require conservation efforts if we are to conserve >~80% of alleles in the species. These findings have important consequences for conservation management that relies on remnant island populations for the preservation of declining species and/or adaptive capacity and highlight some of the ways in which genome-wide SNP data can be combined with environmental data to prioritise conservation management.

### Misplaced faith in island ‘refuges’

Mammal conservation in Australia relies heavily on remnant island populations, for a range of species that have experienced severe declines on the mainland (Legge et al. 2018). In addition, islands on which particular threatening processes are absent are also frequently used as destinations for translocations in lieu of adequate management practices at mainland sites (Abbott 2000; Legge et al. 2018). While the latter situation is not necessarily inappropriate (but see Jolly et al. 2018), the assumption that a species is adequately conserved due to the presence of remnant or abundant island populations could be problematic for two primary reasons. First, many islands or island groups are known to harbour unique lineages of many species of declining mammals, based largely on morphological differences (Van Dyck and Strahan 2008; Van Dyck et al. 2013). In northern Australia, species such as the northern brush-tailed phascogale (*Phascogale pirata*), Butler’s dunnart (*Sminthopsis butleri*), golden bandicoot (*Isoodon auratus*) and northern quoll (*Dasyurus hallucatus*) are now scarce or extinct on the mainland, while remaining extant on one or more islands or archipelago systems. Populations on these islands may be highly differentiated lineages (i.e. separate management units, *sensu* Moritz (1999), and they may also exhibit substantially reduced genetic diversity and loss of adaptive capacity compared to mainland populations (Cardoso et al. 2009). Secondly, we now know that many species are also declining on such islands, mirroring declines on the mainland (Davies et al. 2018). To consider islands as natural refuge habitats/safe havens is not justifiable or appropriate for the conservation of adaptive capacity, and large stable populations will not necessarily persist without active conservation management and planning.

The population genomic structure of the brush-tailed rabbit-rat is characterised by high levels of population differentiation resulting from long periods of isolation between regions. The high level of genomic differentiation we observed between the Tiwi Islands and the rest of the species distribution agrees with morphological evidence suggesting the existence of two Australian subspecies (Kemper and Schmitt 1992; Van Dyck and Strahan 2008). This suggests that conservation activities should consider the Tiwi Islands and the mainland populations as two separate management units. Our conservation prioritisation analysis showed that conserving only the Tiwi Islands populations will not prevent the loss of substantial amounts of allelic diversity and associated adaptive capacity. Conservation benefits were substantially increased by conserving at least two populations, with the greatest benefit achieved by conserving both Melville Island and Cobourg. This is concerning, as there is currently a lack of targeted management specifically designed to ensure the long-term conservation of the brush-tailed rabbit-rat in either of these locations. While intensive fire management is implemented across Melville Island and the Cobourg Peninsula, the extent to which this will conserve species in the long-term remains unclear. As such, brush-tailed rabbit-rat populations in these areas may remain vulnerable to decline. Further work is needed to identify the population trends and status of the brush-tailed rabbit-rat in PNG. However, considering the strong differentiation we observed between the Tiwi Islands and nearby mainland populations, it is likely that the more geographically distant and isolated PNG populations represent a highly differentiated lineage of the species and should be treated as such.

### Implications of population genomic structure for management

Despite being listed in Australia’s Threatened Species Strategy (DoE 2015) as a priority mammal for improving the trajectory of population declines by 2020, very little active conservation management targeted at the brush-tailed rabbit-rat has been undertaken in any region of the species’ range. Continuing declines in this species appear to be occurring in most regions (Davies et al. 2018; von Takach et al. 2020), with inadequate conservation management of the last known population in Australia’s largest national park (Kakadu, e.g., Firth et al. 2005) thought to be extinct. Unfortunately, as no viable tissue samples appear to have ever been collected from the Kakadu region, we are not able to quantify the level of isolation between this population and others on the Australian mainland. However, there is some anecdotal evidence from early reports by naturalists suggesting that population connectivity was considerably greater historically than it has been for the past 50–100 years (Woinarski et al. 2001). The status of the species on Inglis Island also needs to be updated. The brush-tailed rabbit-rat was considered “very abundant” on Inglis Island in the 1990s (Woinarski et al. 1999), with the species known to be present in 2012, but the status and trend of the population since then remains unclear. As this particular island is considered to be free of feral cats, understanding the population dynamics of brush-tailed rabbit-rats on Inglis Island may help to clarify the drivers of mammal population declines in other locations.

One of the values of population genomic analysis for the management of threatened species is the provision of an additional tool for maximising conservation outcomes with available and often limited resources (Green et al. 2018). Decision-making tools are available for the prioritisation of conservation actions; however, few consider genetic or genomic factors, probably because the effect on population viability is not immediate or obvious. In our prioritisation, we assumed equal cost to conserve any given population, resulting in genetic factors primarily influencing the outcome. In reality, actions to conserve this species are unlikely to have equal cost for each population, and considerations of land tenure (National Park, indigenous protected area or privately owned land), current land management and co-benefits for other species, will influence both financial costs and resource availability. Additional factors such as the importance of the species to relevant cultural or interest groups may also require consideration. Incorporating such multidimensional factors into the cost of conserving populations will improve the prioritisation process and stakeholder engagement with conservation actions.

There was a recent (2017) attempt to translocate wild-caught brush-tailed rabbit-rats from the Cobourg Peninsula to Field Island (an island component of Kakadu National Park), despite the species not known to have been on the island historically. However, this translocation did not go ahead as the desired number of individuals (30) to be translocated could not be captured. An earlier attempt to reintroduce brush-tailed rabbit-rats into the Darwin region was also unsuccessful, with four translocation sites (each receiving between 10 and 19 individuals) failing to establish viable populations. Future attempts to translocate or reintroduce this species to Kakadu and other parts of the mainland need to be carefully evaluated, including considering whether individuals from the Cobourg Peninsula are ecologically and genetically most appropriate. While Cobourg populations are likely to be much more genetically similar to the original Kakadu populations than those of the Tiwi Islands, our spatial autocorrelation analysis showed that allele frequencies in individuals separated by more than about 150 km are not significantly correlated. It is possible that populations in the Kimberley region, despite its greater geographic distance, may have historically been less differentiated from the Kakadu region than the Cobourg Peninsula. Investigating the ecological niche of historically occupied locations may help to identify populations that are likely to be most suited/adapted to the environmental conditions in Kakadu National Park. Similarly, prior to any reintroduction operation, the conditions in the receiving environment need to be more generally suitable for the persistence of this species, and this requires concerted effort to manage the environment appropriately. This is most likely to involve ongoing suppression of feral cat and herbivore populations, as well as maintaining suitable fire regimes (i.e. reducing the frequency of high-intensity fires).

### Conservation in the realised niche

Using niche hypervolume analysis, we showed that the niche conditions occupied by the two major lineages of the brush-tailed rabbit-rat are essentially independent of one another. The Tiwi Islands lineage of the species occurs in a highly restricted climatic and topographic set of conditions that are not found across most or all of the species remaining distributed. While we did not investigate the genomic and niche similarity of the Groote Eylandt population, due to a lack of tissue samples from the island, further work on this population would be highly informative. Populations on Groote Eylandt may, similar to the Tiwi Islands, show high levels of genomic differentiation from the mainland, and the environmental conditions occupied on the island may be more similar to the Tiwi Islands than the remaining occupied mainland sites. Comparison of the historical and contemporary niche conditions of the brush-tailed rabbit-rat shows that, at least in the Northern Territory region, the species has broadly contracted towards areas of mild temperatures, low topographic heterogeneity and high moisture availability (von Takach et al. 2020). On the Tiwi Islands, it appears as though high fire frequency has had a big impact on the abundance of local populations (Firth et al. 2010), although the interplay between the effects of fire and other drivers of decline, such as feral cats, is complex (Davies et al. 2017, 2018; Penton et al. 2020). For instance, Radford et al. (2020) found that brush-tailed rabbit-rat abundance (trap success) increased with the increasing extent of early dry season prescribed burning at the Mitchell Plateau. This suggests that low intensity, patchy fire regimes may benefit the species (Davies et al. 2018). The severity of decline exhibited by the brush-tailed rabbit-rat, likely reflects a number of life-history characteristics that contribute to the vulnerability of mainland and island populations. The brush-tailed rabbit-rat has reportedly been most abundant in tall open eucalypt forests with a relatively intact shrub layer and ground cover of grasses (Firth et al. 2006; Davies et al. 2017)—however, areas that are highly fire-prone and with lower shrub cover may promote predation by feral cats (McGregor et al. 2014, 2015; Davies et al. 2017). The brush-tailed rabbit-rat has a highly restricted home range, limited dispersal capacity and often dens in fallen logs that are vulnerable to fire (Firth et al. 2006; Penton et al. 2020). Further, like many old-endemic rodents, the brush-tailed rabbit-rat has a relatively slow reproductive rate with small litter sizes and long gestation periods (Kemper 2020). Thus, to adequately conserve this species, and similarly declining mammals, conservation management needs to provide long-term improvements in habitat suitability by promoting increased vegetation structure and understorey diversity (von Takach et al. 2020b; Radford et al. 2020; Stobo‐Wilson et al. 2020). This likely requires sustained commitment to feral herbivore control, reductions in fire frequency and intensity and feral cat management.

## Conclusion

A range of small mammal species in northern Australia are undergoing severe declines. We have demonstrated that one of these species, the brush-tailed rabbit-rat, has highly distinct lineages between the Tiwi Islands and other populations, and management activities need to aim to conserve multiple populations of the species if we are to conserve its adaptive capacity. This may also be the case for a range of threatened mammals that exhibit similar phylogeographic patterns, and research into the population genomic diversity of other northern Australian species will help to clarify this. Our findings highlight that reliance on islands as natural refuges for the conservation of Australia’s threatened mammal species is likely inappropriate for the long-term conservation of genetic diversity and adaptive capacity. In future decades, if we are able to successfully mitigate both the proximate and ultimate drivers of decline in northern Australia, there will be a need to repopulate vast areas of many species’ former ranges, and understanding patterns of population genomic diversity in the manner presented here will be critical to this management goal. We hope this study emphasises the need for active long-term management of habitats and threatening processes to improve population trajectories across the remaining populations of declining species.

## Supplementary information

Supplemental Material

## Data Availability

All sequencing data have been uploaded to the Oz Mammal Genomics Initiative data portal (https://data.bioplatforms.com/organization/about/bpa-omg) (dataset ID 102.100.100/52622). All scripts and relevant metadata have been uploaded to the Dryad Digital Repository (10.5061/dryad.pc866t1nf).
