# Supplementary material for: Population genomics and conservation management of a declining tropical rodent
Source: Heredity (Edinb). 2021 Mar 4;126(5):763–75. doi: 10.1038/s41437-021-00418-9 (PMC8102610; doi:10.1038/s41437-021-00418-9)
Supplement: Supplementary file 1 — Supplemental Material [file 41437_2021_418_MOESM1_ESM.docx]

**Population genomics and conservation management of a declining tropical rodent**

**Supplementary material**


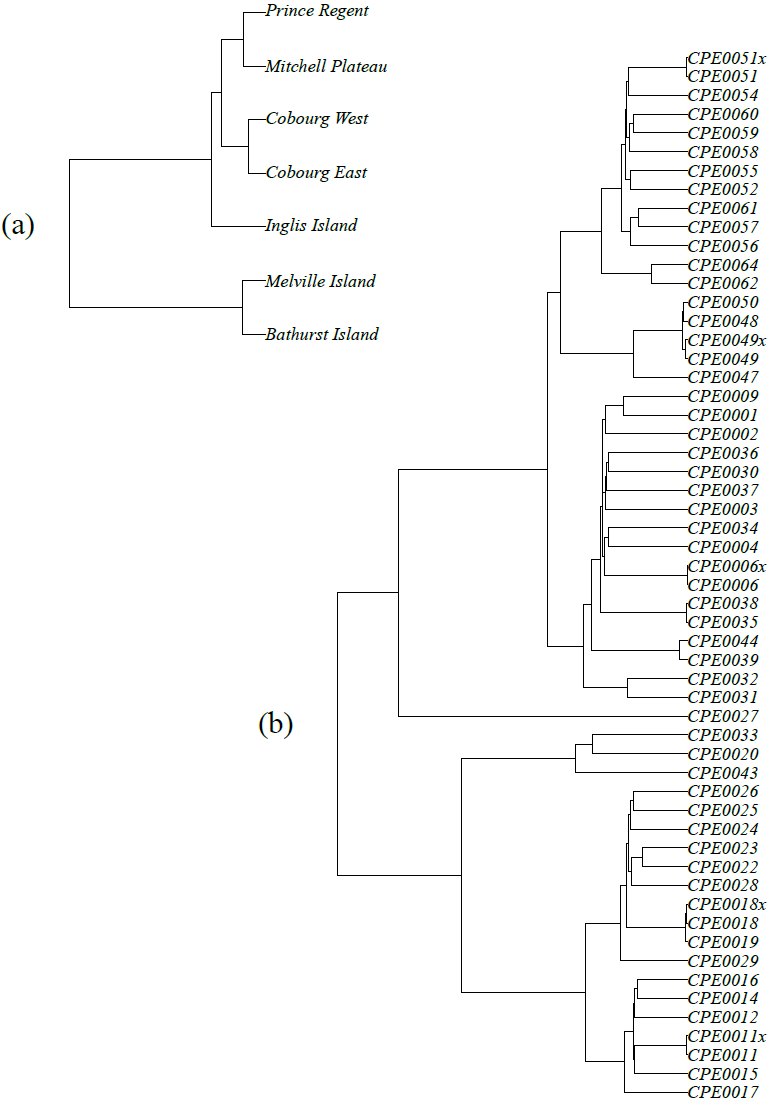


Figure S1. Hierarchical clustering dendrograms representing genetic distance relationships between *Conilurus penicillatus* populations (a) and samples (b). Calculations were made using 11478 single-nucleotide polymorphisms from across the genome. Technical replicates from the field are identified with an ‘x’ after the sample name in (b) and are paired together on branches. Note that these relationships do not necessarily reflect a true phylogeny, which would require further analysis.


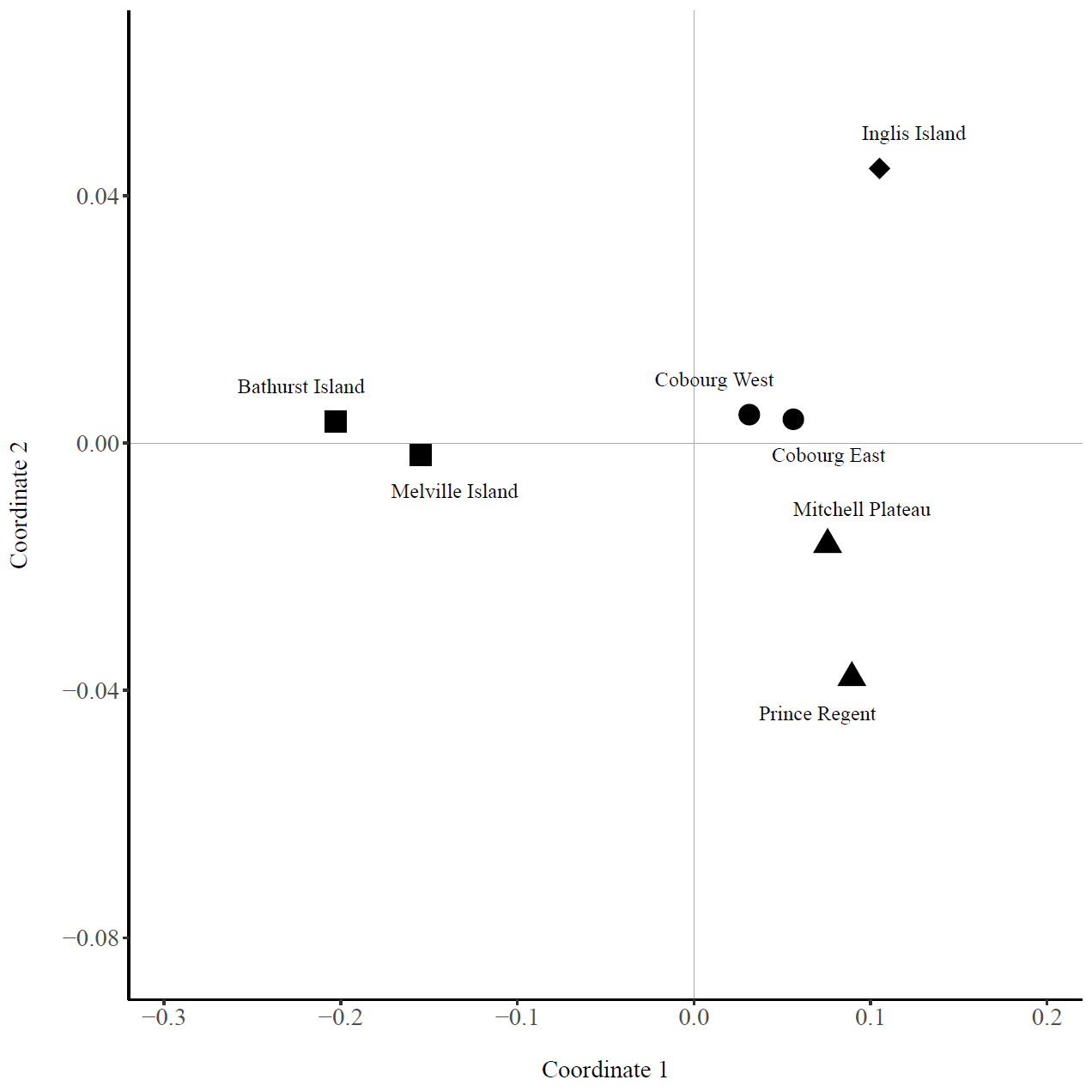


Figure S2. Principal coordinate plot of Nei’s genetic distance between all populations of brush-tailed rabbit-rat (*Conilurus penicillatus*). Broad regions of the species distribution are represented by shapes (squares = Tiwi Islands, triangles = Kimberley region, circles = Cobourg Peninsula, and diamond = Inglis Island). Coordinates 1 and 2 explain 87.1% and 5.8% of the total variance respectively.


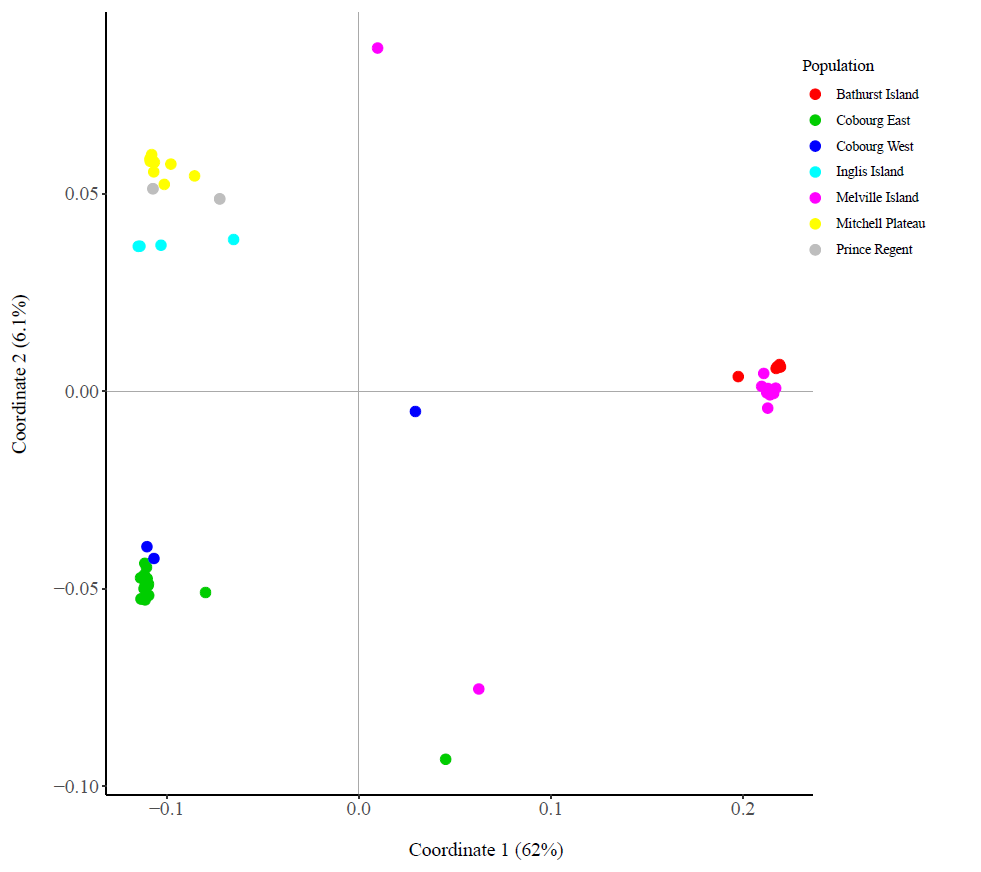


Figure S3. Principal coordinate plot of genetic distance between all 51 individuals of brush-tailed rabbit-rat (*Conilurus penicillatus*). Each population has been given a unique colour. The percentage of the total variance explained by each axis is shown on the axis label.


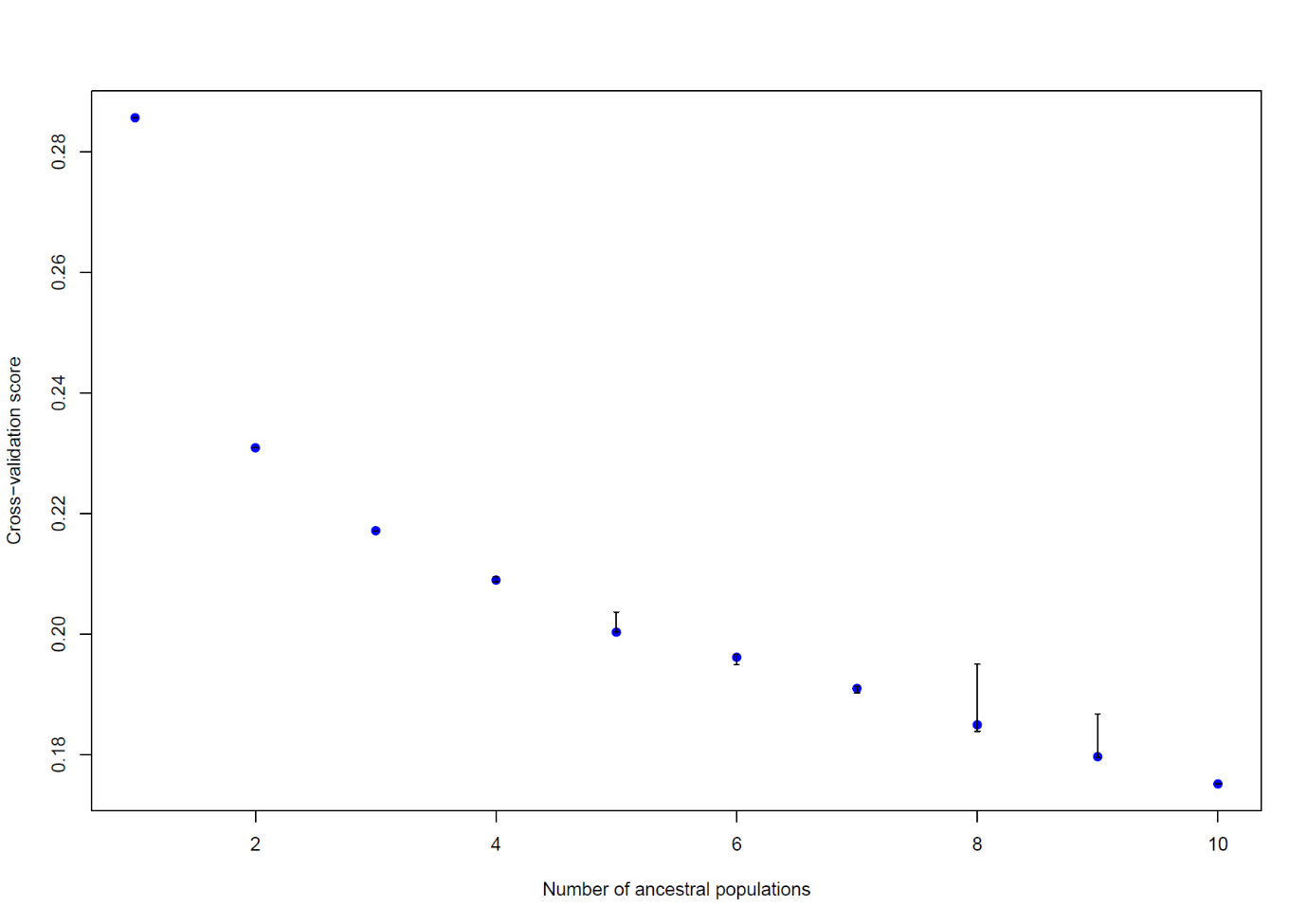


Figure S4. Cross-entropy plot used to identify hierarchical population structuring in the genomic dataset for brush-tailed rabbit-rats (*Conilurus penicillatus*). Lower values of the cross-entropy criterion indicate a better fit to the data. The large drop in cross-entry between 1 and 2 ancestral populations indicates that a *k* value of 2 is well-supported.


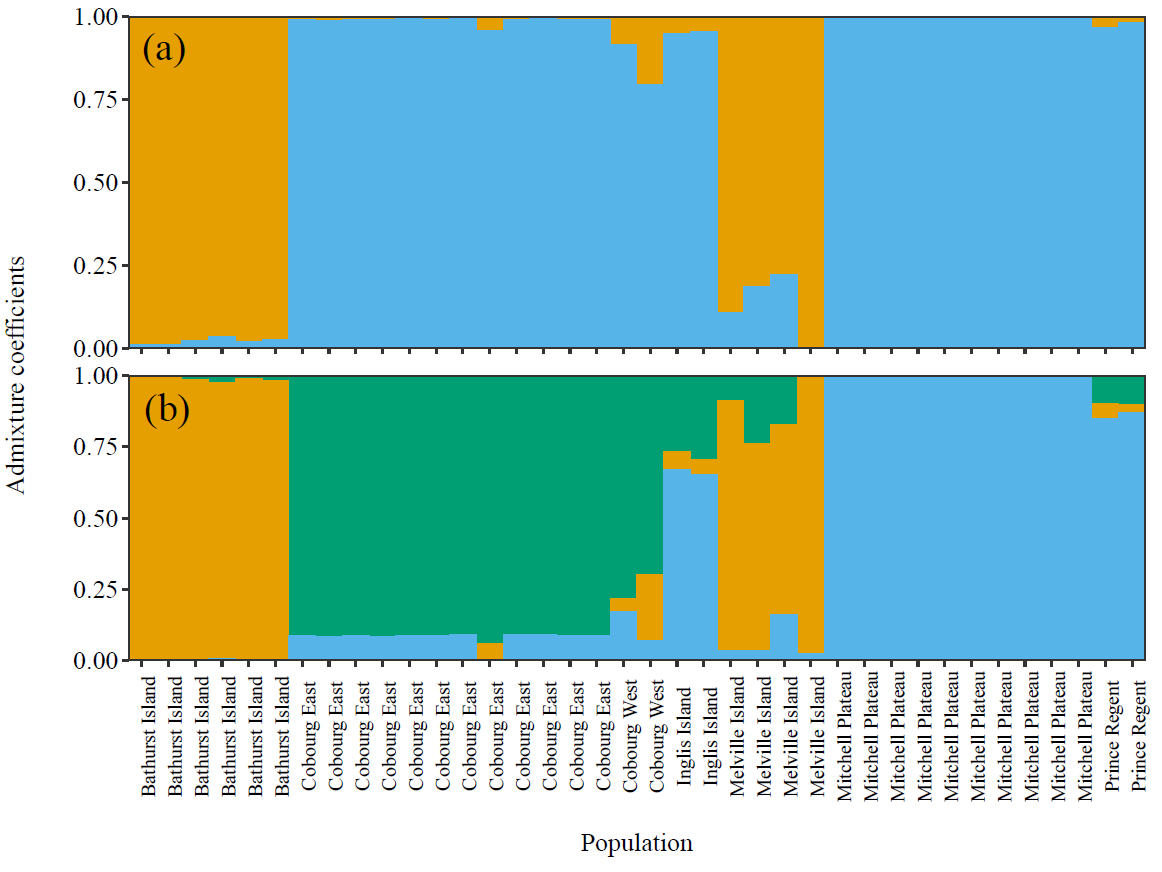


Figure S5. Patterns of population structuring in the brush-tailed rabbit-rat (*Conilurus penicillatus*), **after removal of closely related individuals**. Panels (a) and (b) show the individual admixture coefficients when two or three ancestral genomic clusters are identified, respectively.


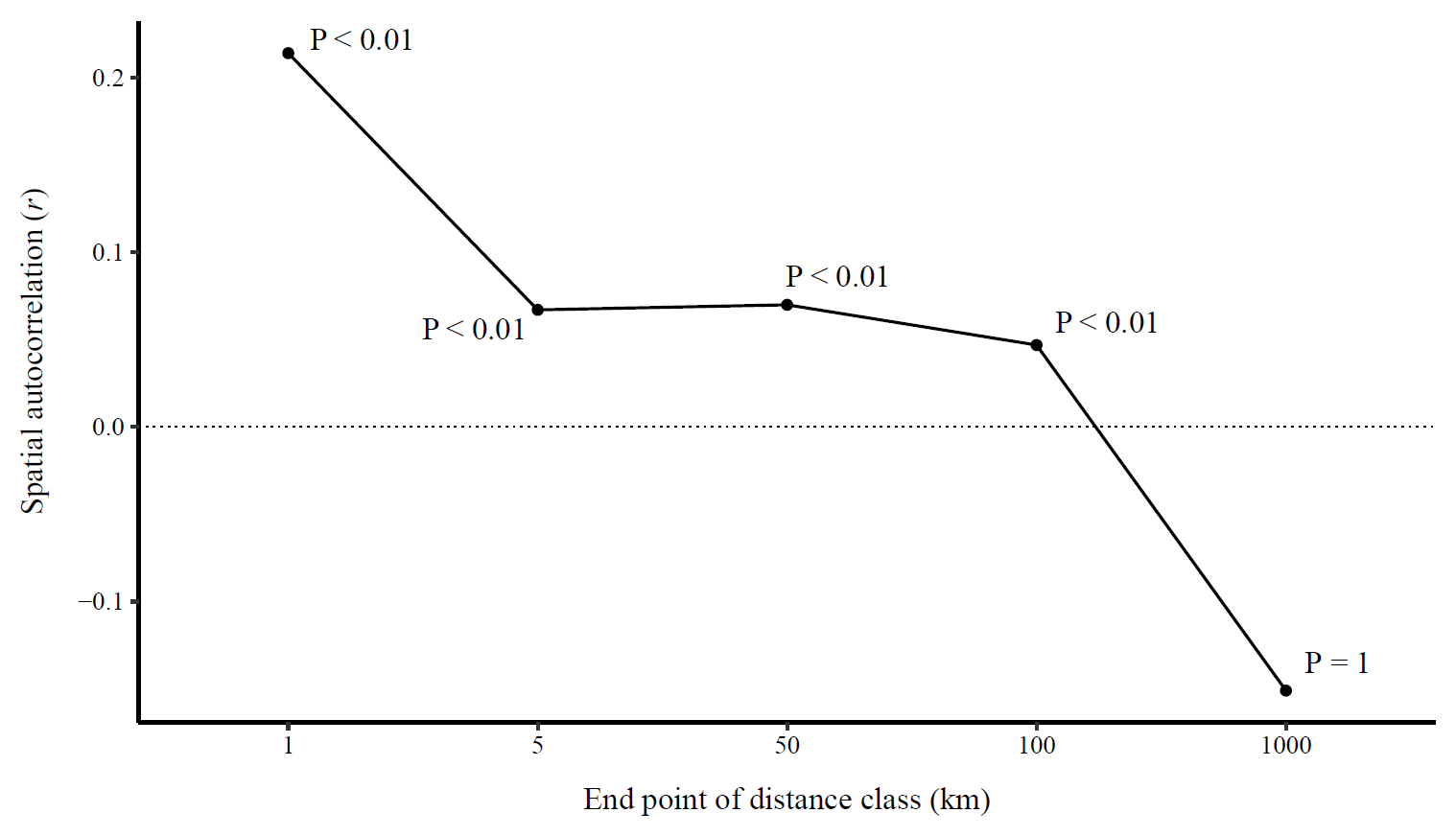


Figure S6. Spatial autocorrelation of multilocus genotypes for individual brush-tailed rabbit-rats (*Conilurus penicillatus*) on mainland Australia at five distance classes. The probability value at each distance class shows the proportion of permuted *r* values greater than the observed value in that distance class, based on 999 permutations of the SNP by sample matrix.


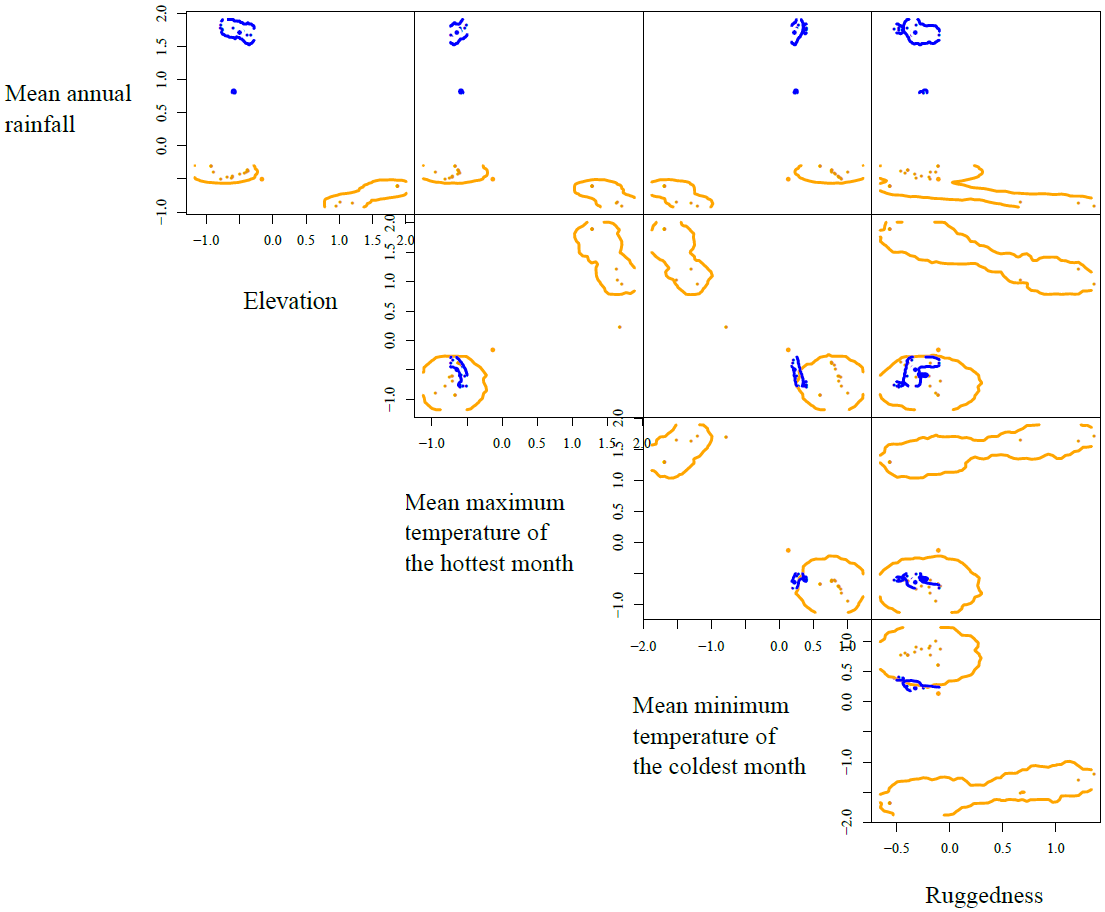


Figure S7. Niche hypervolumes presented as pairwise plots between all genotyped brush-tailed rabbit-rat sampling locations for all five niche dimensions analysed. Colours represent the two lineages that were identified in genome-wide analyses of single-nucleotide polymorphisms, with orange representing the mainland lineage and blue representing the Tiwi Islands lineage. Points represent values of variables at tissue sampling localities. Niche hypervolumes are represented as lines around points.

Table S1. Pairwise population genomic differentiation (*F*_ST_) between all four populations of the brush-tailed rabbit-rat (*Conilurus penicillatus*), **after removal of closely related individuals from the dataset**. All positive *F*_ST_ values are significant at p ≤ 0.001.

|  | Cobourg East | Bathurst Island | Melville Island | Mitchell Plateau |
| --- | --- | --- | --- | --- |
| Cobourg East (n = 12) | 0 |  |  |  |
| Bathurst Island (n = 6) | 0.72 | 0 |  |  |
| Melville Island (n = 4) | 0.40 | 0.19 | 0 |  |
| Mitchell Plateau (n = 10) | 0.29 | 0.78 | 0.47 | 0 |

Table S2. Pairwise population genomic differentiation using a standardised metric (*G*″_ST_) between all four populations of brush-tailed rabbit-rat (*Conilurus penicillatus*) with n ≥ 6. All positive *G*″_ST_ values have p ≤ 0.001.

|  | Cobourg East | Bathurst Island | Melville Island | Mitchell Plateau |
| --- | --- | --- | --- | --- |
| Cobourg East | 0 |  |  |  |
| Bathurst Island | 0.78 | 0 |  |  |
| Melville Island | 0.67 | 0.25 | 0 |  |
| Mitchell Plateau | 0.31 | 0.84 | 0.73 | 0 |

Table S3. Pairwise population genomic differentiation using a standardised metric (*G*″_ST_) between all four populations of brush-tailed rabbit-rat (*Conilurus penicillatus*), **after removal of closely related individuals from the dataset**. All positive *G*″_ST_ values have p ≤ 0.001.

|  | Cobourg East | Bathurst Island | Melville Island | Mitchell Plateau |
| --- | --- | --- | --- | --- |
| Cobourg East | 0 |  |  |  |
| Bathurst Island | 0.78 | 0 |  |  |
| Melville Island | 0.49 | 0.25 | 0 |  |
| Mitchell Plateau | 0.31 | 0.84 | 0.56 | 0 |

Table S4. Population genetic parameters for the four populations of brush-tailed rabbit-rat (*Conilurus penicillatus*) where n ≥ 6. Parameters include the number of samples (*N*), number of alleles (*A*), effective number of alleles (*A*_E_), observed heterozygosity (*H*_O_), expected heterozygosity (*H*_E_), Wright’s inbreeding coefficient (*F*_IS_), and the locus polymorphic index (*P*_E_).

| Population | *N* | *A* | *A*_E_ | *H*_E_ | *H*_O_ | *F*_IS_ | *P*_E_ |
| --- | --- | --- | --- | --- | --- | --- | --- |
| Bathurst Island | 6 | 1.16 | 1.09 | 0.06 | 0.06 | 0.02 | 0.05 |
| Cobourg East | 15 | 1.51 | 1.14 | 0.10 | 0.09 | 0.03 | 0.09 |
| Melville Island | 11 | 1.55 | 1.17 | 0.12 | 0.09 | 0.31 | 0.12 |
| Mitchell Plateau | 10 | 1.22 | 1.10 | 0.06 | 0.06 | 0.02 | 0.06 |

Table S5. Population genetic parameters for the four populations of brush-tailed rabbit-rat (*Conilurus penicillatus*), **after removal of closely related individuals from the dataset**. Parameters include the number of samples (*N*), number of alleles (*A*), effective number of alleles (*A*_E_), observed heterozygosity (*H*_O_), expected heterozygosity (*H*_E_), Wright’s inbreeding coefficient (*F*_IS_), and the locus polymorphic index (*P*_E_).

| Population | *N* | *A* | *A*_E_ | *H*_E_ | *H*_O_ | *F*_IS_ | *P*_E_ |
| --- | --- | --- | --- | --- | --- | --- | --- |
| Bathurst Island | 6 | 1.16 | 1.09 | 0.06 | 0.06 | 0.02 | 0.05 |
| Cobourg East | 12 | 1.50 | 1.14 | 0.10 | 0.09 | 0.03 | 0.09 |
| Melville Island | 4 | 1.53 | 1.30 | 0.21 | 0.13 | 0.32 | 0.18 |
| Mitchell Plateau | 10 | 1.22 | 1.10 | 0.06 | 0.06 | 0.02 | 0.06 |
